# Effect of iron supplementation in patients with heart failure and iron deficiency: A systematic review and meta-analysis

**DOI:** 10.1016/j.ijcha.2021.100871

**Published:** 2021-09-14

**Authors:** Naser Yamani, Aymen Ahmed, Priyanka Gosain, Kaneez Fatima, Ali Tariq Shaikh, Humera Qamar, Izza Shahid, Muhammad Sameer Arshad, Talal Almas, Vincent Figueredo

**Affiliations:** a Department of Medicine, John H Stroger Jr. Hospital of Cook County, Chicago, IL, USA; b Department of Medicine, Dow University of Health Sciences, Karachi, Pakistan; c Department of Medicine, Memorial Healthcare System, Pembroke Pines, USA; d Department of Internal Medicine, Dow University of Health Sciences, Karachi, Pakistan; e Department of Medicine, Khaja Bandanawaz Institute of Medical Sciences Gulbarga, India; f Department of Medicine, Ziauddin Medical University, Karachi, Pakistan; g Department of Medicine, RCSI University of Medicine and Health Sciences, Dublin, Ireland; h Department of Cardiology, St.Mary Medical Center, Langhorne, PA, USA

**Keywords:** Oral, Intravenous, Iron sucrose, Ferric carboxymaltose, Heart failure, Iron deficient

## Abstract

**Background:**

The effectiveness of oral and intravenous iron supplementation in reducing the risk of mortality and hospitalizations in HF patients with iron deficiency is not well-established.

**Methods:**

A thorough literature search was conducted across 2 electronic databases (Medline and Cochrane Central) from inception through March 2021. RCTs assessing the impact of iron supplementation on clinical outcomes in iron deficient HF patients were considered for inclusion. Primary end-points included all-cause mortality and HF hospitalization. Evaluations were reported as odds ratios (ORs) or risk ratios (RRs) with 95% confidence intervals (CI) and analysis was performed using a random effects model. I^2^ index was used to assess heterogeneity.

**Results:**

From the 2599 articles retrieved from initial search, 10 potentially relevant studies (n = 2187 patients) were included in the final analysis. Both oral (OR: 0.93; 95% CI: 0.08–11.30; p = 0.951) and intravenous (OR: 0.97; 95% CI: 0.73–1.29; p = 0.840) iron supplementation did not significantly reduce all-cause mortality. However, intravenous iron supplementation significantly decreased the rates of overall (OR: 0.52; 95% CI: 0.33–0.81; p = 0.004) and HF (OR: 0.42; 95% CI: 0.22–0.80; p = 0.009) hospitalizations. In addition, intravenous ferric carboxymaltose therapy significantly reduced the time to first HF hospitalization or cardiovascular mortality (RR = 0.70; 95% CI = 0.50–1.00; p = 0.048), but had no effect on time to first cardiovascular death (RR: 0.94; 95% CI: 0.70–1.25; p = 0.655).

**Conclusion:**

Oral or intravenous iron supplementation did not reduce mortality in iron deficient HF patients. However, intravenous iron supplementation was associated with a significant decrease in overall and HF hospitalizations.

## 1 Introduction

Iron deficiency is recognized as an important comorbidity and independent predictor of outcomes in patients with acute and chronic heart failure (HF) [1]. It is one of the most prevalent (approximately 50%) concomitant disorders present in HF patients, and is associated with poor prognosis, reduced physical well-being, exercise intolerance, repeated hospitalizations and a subsequent increase in mortality, regardless of the presence of anemia [2], [3], [4], [5], [6]. Iron supplementation can potentially improve quality of life and confer greater survival benefits in HF patients with iron deficiency [7]. While oral iron intake has been contraindicated mainly due to adverse gastrointestinal side-effects, intravenous iron supplementation has arisen as a potential therapeutic agent in HF patients, which may administer gradational advantage towards hospitalization and mortality outcomes [8]. The updated American College of Cardiology Foundation/American Heart Association (ACCF/AHA) guidelines and the European Society of Cardiology (ESC) guidelines recommend the use of intravenous iron supplementation in HF patients (NHYA Class II and III) with iron deficiency to alleviate their functional status and quality of life [9], [10]. However, the effectiveness of both oral and intravenous iron supplementation on clinical outcomes in iron-deficient HF patients remains unclear due to lack of reliable evidence and contrasting findings in prior clinical trials and meta-analyses [11], [12], [13], [14]. Therefore, in light of the inconsistent results, we conducted a meta-analysis to evaluate the efficacy of iron supplementation in reducing mortality and hospitalizations in iron deficient HF patients.

## 2 Methods

This systematic review and meta-analysis has been reported in concordance with guidelines provided by the Preferred Reporting Items for Systematic Reviews and Meta-Analysis statement (PRISMA) [15]. Approval from the institutional review board was not required since the data was publicly available.

Informed consent was obtained from each patient included in the studies and the study protocol conforms to the ethical guidelines of the 1975 Declaration of Helsinki as reflected in a priori approval by the institution's human research committee.

### 2.1 Literature search strategy and data sources

We systematically searched two databases (Medline and Cochrane Central) for randomized controlled trials assessing the impact of intravenous or oral iron supplementation in iron deficient HF patients from inception through March 2021, without any time or language restrictions. Additional sources included bibliographies of review articles, original studies and relevant editorials. Mesh terms along with Boolean operators were used to devise an effective search strategy for each database (Table S1).

### 2.2 Study selection

All articles retrieved from the systematic search were exported to EndNote Reference Manager (Version X7.5; Clarivate Analytics, Philadelphia, Pennsylvania) where duplicates were sought and removed. The remaining articles were then assessed at title and abstract level by two independent investigators (NY and AA), after which full text were read to confirm relevance. Any disagreements were resolved by mutual discussion with a third investigator (VF). The following pre-defined inclusion criteria was used: (1) studies which investigated the effects of oral or intravenous iron supplementation on clinical outcomes in iron deficient HF, (2) were placebo-controlled double-blinded randomized controlled trials, and (3) had a minimum follow-up duration of 12 weeks. Data from smaller unpublished trials, post-hoc analyses or individual patient data analyses of trials comparing iron supplementation with placebo were also considered for inclusion.

### 2.3 Data extraction and outcomes of Interest

Two investigators (NY and AA) autonomously extracted data from the selected studies on pre-specified collection forms. Apart from baseline trial and patient characteristics, data were abstracted for clinical outcomes namely all-cause mortality, overall and HF hospitalization, time to first HF hospitalization or cardiovascular death and time to first cardiovascular death. The potential risk of bias of the short-listed RCTs was evaluated using the modified Cochrane Collaboration’s risk of bias tool [16].

### 2.4 Statistical analyses

All statistical analyses were performed using Open Meta-Analyst (V10.10 CEBM @ Brown, New Jersey, USA). The results for dichotomous outcomes were presented as odds ratios (ORs) with 95% confidence intervals (CIs) while risk ratios (RRs) with 95% CIs were used for time-to-event outcomes. Logit transformation for both ORs and RRs was carried out and meta-analysis was done using a random effects model [17]. Inclusion of a limited number of studies did not permit the evaluation of a publication bias [18]. The I^2^ statistic was used to evaluate heterogeneity across studies, and a value of I2 = 25%-50% was considered mild, 50%-75% as moderate and I2 > 75% as severe [19]. A p-value < 0.05 was considered statistically significant in all cases.

## 3 Results

### 3.1 Literature search

**Fig. 1:**
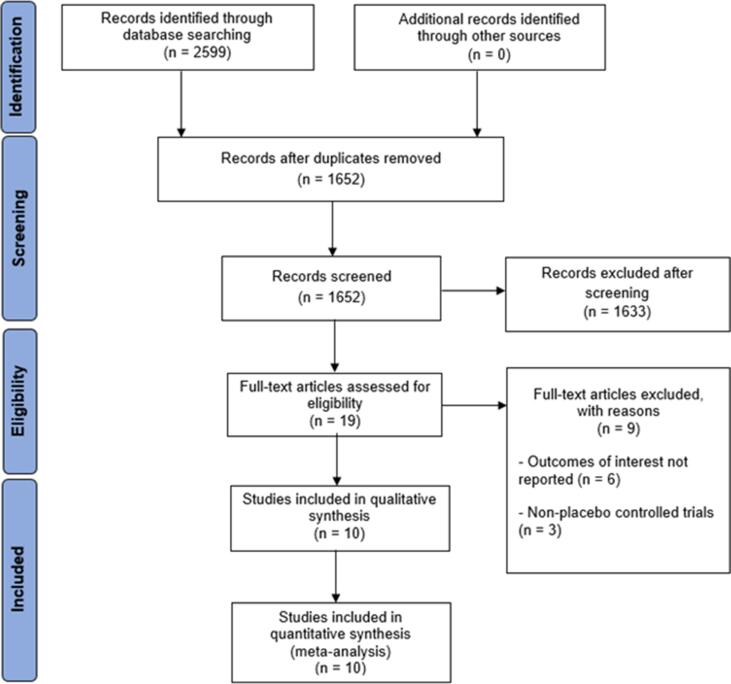
PRISMA flowchart outlining literature search.

The initial literature search yielded 2,599 potentially relevant articles. After applying the predetermined eligibility criteria, 10 studies were selected for inclusion in this meta-analysis [11], [12], [13], [14], [20], [21], [22], [23], [24]. The PRISMA flowchart summarizes the results of our literature search (Fig. 1).

### 3.2 Study characteristics and quality assessment

Our short-listed studies included 2187 patients (56.8% males; mean age-68.6 years) over a median follow-up of 18 weeks. Only HF patients with reduced LVEF were recruited in all trials, with a cut-off for LVEF ranging from 35% to ≤50% at baseline. The target hemoglobin was not defined in most included trials. However, the goal was to replenish iron levels above the normal cut-off. Baseline demographics of the included studies in this meta-analysis are outlined in Table 1.

**Table 1:** Baseline characteristics of the included trials.

Trial/patient characteristics	AFFIRM-AHF	CONFIRM-HF	EFFICACY-HF	FAIR-HF	FER-CARS-01	IRON-HF	FERRIC-HF	McCullough et al	Toblli et al	Arutyunov et al
n (iron therapy/placebo)	558/550	150/151	20/14	20/14	304/155	17/6	24/11	40/41	20/20	57/15
Iron therapy	IV FCM	IV FCM	IV FCM	IV FCM	IV FCM	IV Iron Sucrose and Oral Ferrous Sulfate	IV Iron Sucrose	Oral Ferric Citrate	IV Iron Sucrose	IV Iron Sucrose and FCM
Follow-up (weeks)	52	52	24	24	12	12	18	16, 12	24	14
Study type	RCT	RCT	RCT	RCT (data for analysis taken from individual patient data meta-analysis by Anker et al)	RCT (data for analysis taken from individual patient data meta-analysis by Anker et al)	RCT	RCT	Individual patient data post hoc analysis of 2 trials	RCT	RCT
Study design	Multi-center	Multi-center	Multi-center	Multi-center	Multi-center	Multi-center	Multi-center	Multi-center	NA	NA
Age (years)	71/71	69/70	NA	68/67	NA	66/69	64/62	65/68	76/74	NA
Males (%)	56/54	55/51	NA	48/45	NA	71/67	71/73	52/39	NA	NA
NYHA Class	NA	2.5/2.4	NA	2.8/2.8	NA	NA	2.5/2.4	NA	2.9/2.9	NA
TSAT (%)	15/14	20/18	NA	18/17	NA	19/14	20/21	20/20	0.2/0.2	NA
NT-proBNP (pg/ml)	4743/4684	2511/2600	NA	NA	NA	NA	NA	NA	256/268	NA
Ischemic HF (%)	48/47	83/83	NA	81/79	NA	29/67	NA	38/61	NA	NA
LVEF (%)	33/33	37/37	NA	32/33	NA	27/31	30/29	NA	31.3/30.8	NA
Ferritin (μg/L)	84/89	57/57	NA	53/60	NA	NA	62/88	NA	73/74	NA
Anemia (%)	53/57	53/48	NA	65/61	NA	NA	NA	NA	NA	NA
Hemoglobin (g/dl)	12.3/12.1	12.4/12.4	NA	11.9/11.9	NA	11.2/10.9	12.6/12.2	10.6/10.5	10.3/10.2	NA
HF severity and status	Hospitalization for acute HF, LVEF < 50%,	Ambulatory, NYHA class II/III, systolic CHF with ID	Ambulatory, NYHA class II/III, systolic CHF with ID	Ambulatory, NYHA class II/III, systolic CHF with ID	Ambulatory, NYHA class II/III, systolic CHF with ID	Stable ambulatory HF patients, NHYA class II-IV, LVEF < 40%, with anemia	Symptomatic CHF with ID, NHYA class II/III, LVEF < 45%	HF patients with iron-deficiency anemia	HF patients with ID, NHYA class II-IV, LVEF < 35%	CHF patients
Target Hemoglobin (g/dL)	NA	NA	NA	NA	NA	> 12 (females), >13 (males)	NA	≥10 increase	NA	NA

FAIR-HF, Ferinject Assessment in Patients with Iron Deficiency and Chronic Heart Failure; CONFIRM-HF, Ferric CarboxymaltOse evaluatioN on perFormance in patients with IRon deficiency in coMbination with chronic Heart Failure; AFFIRM-AHF, A Randomised, Double-blind Placebo Controlled Trial Comparing the Effect of Intravenous Ferric Carboxymaltose on Hospitalisations and Mortality in Iron Deficient Subjects Admitted for Acute Heart Failure; FERRIC-HF, Ferric Iron Sucrose in Heart Failure; FCM, ferric carboxymaltose; HF, heart failure; CHF, chronic heart failure; NYHA, New York Heart Association; ID, iron deficiency; LVEF, left ventricular ejection fraction; RCT, randomized controlled trial; TSAT, transferrin saturation; n, number of patients; NA, not available.

Seven (FAIR-HF [Ferinject Assessment in Patients with Iron Deficiency and Chronic Heart Failure], CONFIRM-HF [Ferric CarboxymaltOse evaluatioN on perFormance in patients with IRon deficiency in coMbination with chronic Heart Failure], AFFIRM-AHF [A Randomised, Double-blind Placebo Controlled Trial Comparing the Effect of Intravenous Ferric Carboxymaltose on Hospitalisations and Mortality in Iron Deficient Subjects Admitted for Acute Heart Failure], FERRIC-HF [Ferric Iron Sucrose in Heart Failure], IRON-HF, Toblli et al and Arutyunov et al) of our included studies had an overall low risk of bias (Figure S1), whereas the risk of bias for the remaining three studies could not be ascertained due to the following reasons; (i) for two trials (EFFICACY-HF and FER-CARS-01), study level results have not been published yet hence we extracted results for these trials from the individual patient data meta-analysis published by Anker et al, (ii) two trials were not conducted on HF populations exclusively hence they were not directly included in analysis, however, their results specific to iron-deficient HF patients were extracted from the post-hoc pooled analysis based on individual patient data of these two trials published by McCullough et al.

### 3.3 Meta-analysis Results:

**Fig. 2:**
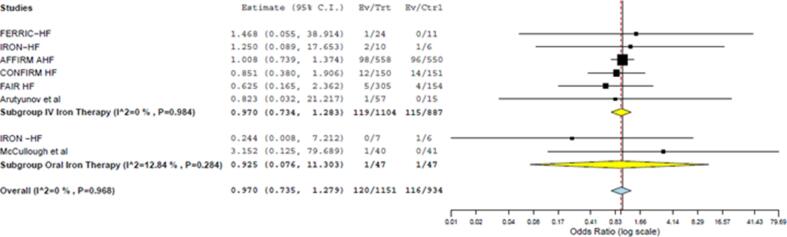
Forest plot comparing the effect of intravenous (p = 0.832) and oral iron (p = 0.951) supplementation on all-cause mortality when compared with placebo (p = 0.828).

**Fig. 3:**
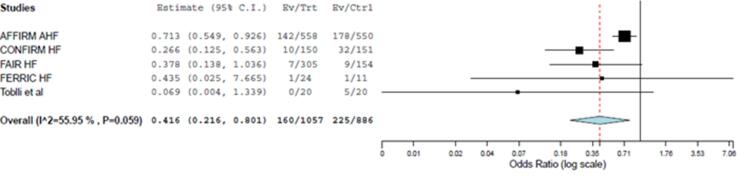
Forest plots displaying the effect of intravenous iron on heart failure hospitalization when compared with placebo (p = 0.009).

**Fig. 4:**
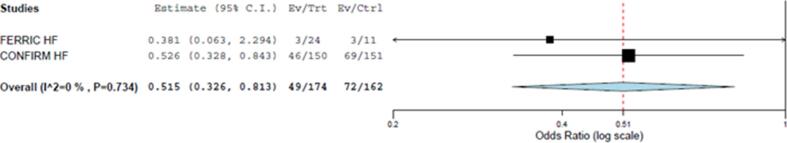
Forest plot displaying the effect of intravenous iron supplementation on hospitalization when compared with placebo (p = 0.004).

**Fig. 5:**
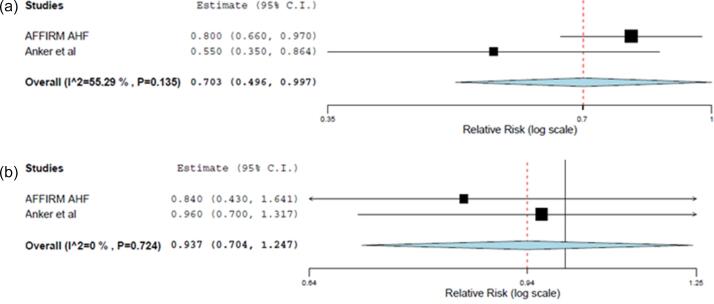
(a) and (b) Forest plots comparing the effect on (a) time to first heart failure hospitalizations or cardiovascular death (p = 0.048) and (b) time to first cardiovascular death (p = 0.655) between intravenous FCM and control group.

Fig. 2, Fig. 3, Fig. 4, Fig. 5 display the effects of iron supplementation on clinical outcomes in patients with HF and iron deficiency when compared with placebo.

#### 3.3.1 All-cause mortality

Seven studies reported the effect of iron supplementation on all-cause mortality. Iron supplementation in iron-deficient HF patients had no significant impact on all-cause mortality (OR: 0.97; 95% CI: 0.74–1.28; p = 0.828) when compared with placebo (Fig. 2). Our results were consistent upon subgroup analysis by type of iron supplementation. Neither intravenous (OR: 0.97; 95% CI: 0.73–1.29; p = 0.840) nor oral iron therapy (OR: 0.93; 95% CI: 0.08–11.30; p = 0.951) significantly reduced all-cause mortality when compared with placebo (Fig. 2).

#### 3.3.2 HF hospitalization

Five studies evaluated the effect of intravenous iron treatment on HF hospitalization. Intravenous iron supplementation significantly reduced the incidence of HF hospitalization (OR: 0.42; 95% CI: 0.22–0.80; p = 0.009) when compared with placebo (Fig. 3). No trials evaluating the effect of oral iron therapy on HF hospitalization were available.

#### 3.3.3 Hospitalization

Only two studies reported on a general hospitalization outcome. Intravenous iron supplementation significantly reduced the incidence of hospitalization (OR: 0.52; 95% CI: 0.33–0.81; p = 0.004) in HF patients with iron deficiency when compared with placebo (Fig. 4). There were no trials assessing the impact of oral iron treatment on hospitalization in iron-deficient HF patients.

#### 3.3.4 Time to first event outcomes

Intravenous iron supplementation by ferric carboxymaltose (FCM) was found to have a statistically significant benefit on the time to first HF hospitalization or cardiovascular mortality (RR = 0.70; 95% CI = 0.50–1.00; p = 0.048) when compared with placebo (Fig. 5a). However, intravenous FCM supplementation did not significantly reduce the time to first cardiovascular death (RR: 0.94; 95% CI: 0.70–1.25; p = 0.655) (Fig. 5b). The effect of oral or other intravenous iron therapies on the time to first event outcomes was not evaluated in any trial.

## 4 Discussion

Our meta-analysis outlines various key findings. First, intravenous or oral iron supplementation has no effect on all-cause mortality in iron-deficient HF patients. Second, intravenous iron supplementation strongly reduces the incidence of hospitalization due to HF as well as overall hospitalizations when compared with placebo. Third, beneficial effects on time to first HF hospitalization or cardiovascular mortality are observed with the usage of intravenous FCM compared with placebo. However, no distinct benefit of intravenous FCM was noted on time to first cardiovascular death.

Our findings concur with prior under-powered meta-analyses and the recent large AFFIRM-AHF trial which have demonstrated similar hospitalization benefits but no notable reduction in all-cause or cardiovascular deaths with iron supplementation in HF patients with iron deficiency [11], [12], [13], [14]. This updated meta-analysis with a larger sample size confirms the existing findings in clinical trials and meta-analyses due to usage of iron therapy in iron-deficient HF patients.

HF has been established as a global pandemic with the 5-year survival rate remaining poor [25]. Unfortunately, our analysis did not reveal any beneficial effect of oral or intravenous iron supplementation on all-cause mortality in HF patients with iron deficiency. This finding might be due to short follow-up durations in most trials whereby the event numbers for mortality remain low. In addition, our results are largely driven by the well-powered AFFIRM-AHF trial and it cannot be ignored that the death rate in this trial might have been influenced by the concomitant existence of COVID-19 disease in some patients. However, our results not reaching statistical power even after meta-analysis indicate that iron deficiency is a risk marker, and not a risk factor for mortality in HF. This means that iron deficiency predicts poor outcomes in HF patients but does not cause them. Underlying pathophysiological processes causing iron deficiency are more likely to be responsible for adverse outcomes in iron-deficient HF patients. Correction of iron deficiency alone using oral or intravenous iron supplementation does not fix the background complications and hence does not lead to a significant mortality benefit. However, small sample sizes and low event rates in most included trials warrant the need for large trials over longer follow-up durations to be conducted in the future.

The risk of HF induced hospitalizations also remains high, despite the significant advances in prevention and therapy [26], [27], [28]. Borderline blood pressure limiting up-titration of appropriate dosages and hyperkalemia often lead to these therapies not being well tolerated [29], [30], [31]. In addition, excessive inhibition of compensatory neuroendocrine systems through medical therapy has been associated with adverse outcomes [23]. Readmission rates stay elevated despite patients receiving guideline directed medical therapy suggesting that other measures need to be considered for improving the overall prognosis of HF patients [32], [33]. Our study showed that intravenous iron supplementation compared with placebo was noted to have a positive impact on hospitalizations due to HF as well as overall hospitalization. Similar findings have been noted in preceding studies where intravenous iron therapy has played a significant role in lowering the risk of HF hospitalizations irrespective of the presence of anemia [34]. Hence, alongside other modern drugs, intravenous iron supplementation can be considered as a potential therapeutic agent for alleviating the global hospitalization burden caused by HF.

Iron deficiency in HF has been defined as serum ferritin concentration < 100 μg/l, or ferritin concentration between 100 and 300 μg/l, along with transferrin saturation (TSAT) of < 20% [8]. It is largely prevalent in patients with HF and has important quality of life and prognostic inferences. However, it often goes unnoticed as the symptoms related to iron deficiency in HF are not clearly evident and an evaluation of iron parameters is the only possible means of its diagnosis. Iron deficiency causes imbalance in hematopoiesis and impairs mitochondrial function, leading to deterioration of heart failure and worsening of the overall prognosis [1], [2]. One of the most common reasons for iron deficiency is renal anemia which is largely prevalent in patients with HF [35], [36], [37]. Iron supplementation combined with erythropoiesis-stimulating agents has been shown to confer benefits in the achievement of target hemoglobin levels in patients with renal anemia and chronic kidney disease (CKD) [37]. Moreover, hypoxia-inducible factor-prolyl hydroxylase (HIF-PF) inhibitors have emerged as beneficial therapeutic agents for the treatment of renal anemia. *Lentini S. et al* showed the potential benefit of combined intake of iron supplements and HIF-PF inhibitors in patients with renal anemia [38]. Therefore, iron therapy along with other agents like HIF-PF inhibitors in iron-deficient HF patients with concomitant renal anemia or CKD may prove to be helpful. However, widespread usage of iron supplementation in this population warrants the need for greater evidence from well-powered clinical trials in future.

Intravenous iron supplementation in iron deficient HF patients has been recommended by current guidelines [9], [10]. While different intravenous iron therapies (iron isomaltose, iron (III) gluconate, iron sucrose, ferumoxytol) exist, the use of intravenous FCM has been widely tested and recommended for improving prognosis in iron deficient HF patients [8], [39]. Various studies have demonstrated that intravenous FCM improves physical well-being, functional status and quality of life and confers hospitalization benefits in HF patients with iron deficiency [40], [41]. In addition, our results signify that intravenous FCM reduces the time to first HF hospitalization or cardiovascular death. These improvements continue to exist even in patients without anemia suggesting that reduced hemoglobin level is merely a consequence of iron diminution.

A considerable number of trials have evaluated the effect of intravenous iron supplementation on clinical outcomes in iron deficient HF patients. However, data regarding the effect of oral iron supplementation on clinically relevant outcomes namely mortality and hospitalizations remains limited. This is likely due to the fact that oral iron treatment has been found to be associated with adverse gastrointestinal events. Additionally, the IRONOUT HF (Oral Iron Repletion Effects On Oxygen Uptake in Heart Failure) trial demonstrated no beneficial effect of oral iron supplementation on time to death or cardiovascular hospitalization or on primary (peak oxygen uptake) or secondary end-points (NT-proBNP levels and 6-minute walk test) [42]. However, in order to address the lack of evidence with oral iron supplementation in HF, well-powered trials in future need to assess clinical outcomes over longer follow-ups. Nonetheless, intravenous iron supplementation is a guideline recommended therapy and has proven to be a better option with considerable hospitalization and quality of life benefits.

## 5 Limitations

This meta-analysis is subject to several limitations. Firstly, the optimum dose of intravenous or oral iron supplementation in HF patients could not be established due to the lack of availability of data. Secondly, the trials included in our analysis were based on patients with acute (AFFIRM-AHF) as well as chronic HF, which might have contributed to considerable heterogeneity in our findings. Thirdly, the included studies mainly involved HF patients with reduced ejection fraction. Hence, our findings may not be generalizable over HF patients with preserved ejection fraction. Fourthly, the effects of oral iron supplementation on clinical outcomes could not be evaluated properly due to lack of data. More trials need to be conducted in future to assess the impact of oral iron therapy on mortality and hospitalization outcomes. Lastly, the results of our meta-analysis were mostly determined by the well-powered AFFIRM-AHF trial since other included trials were usually smaller or underpowered. Present ongoing large trials [IRONMAN (NCT02642562), HEART-FID (NCT03037931), FAIR-HF2 (NCT03036462), FAIR HFpEF (NCT03074591)] will affirm the effectiveness of intravenous iron supplementation in iron deficient HF patients.

## 6 Conclusion

Intravenous or oral iron supplementation in iron-deficient HF patients has no effect on all-cause mortality. However, intravenous iron supplementation strongly reduces the incidence of overall as well as HF hospitalizations. Additionally, intravenous FCM supplementation has also been found to reduce the time to first HF hospitalization or cardiovascular death. Larger prospective randomized controlled trials in future will help validate the effectiveness of iron supplementation on clinical outcomes in HF patients with iron deficiency.

## Declaration of Competing Interest

The authors report no relationships that could be construed as a conflict of interest.

## References

[1] Ebner N., von Haehling S. (2019). Why is iron deficiency recognised as an important comorbidity in heart failure?. Card. Fail. Rev..

[2] Anand I.S., Gupta P. (2018). Anemia and iron deficiency in heart failure: current concepts and emerging therapies. Circulation.

[3] Klip I.T., Comin-Colet J., Voors A.A., Ponikowski P., Enjuanes C., Banasiak W., Lok D.J., Rosentryt P., Torrens A., Polonski L., van Veldhuisen D.J., van der Meer P., Jankowska E.A. (2013). Iron deficiency in chronic heart failure: an international pooled analysis. Am. Heart J..

[4] Comin-Colet J., Enjuanes C., Gonzalez G., Torrens A., Cladellas M., Merono O., Ribas N., Ruiz S., Gomez M., Verdu J.M., Bruguera J. (2013). Iron deficiency is a key determinant of health-related quality of life in patients with chronic heart failure regardless of anaemia status. Eur. J. Heart Fail..

[5] Jankowska E.A., Rozentryt P., Witkowska A., Nowak J., Hartmann O., Ponikowska B., Borodulin-Nadzieja L., von Haehling S., Doehner W., Banasiak W., Polonski L., Filippatos G., Anker S.D., Ponikowski P. (2011). Iron deficiency predicts impaired exercise capacity in patients with systolic chronic heart failure. J. Card Fail..

[6] Okonko D.O., Mandal A.K., Missouris C.G., Poole-Wilson P.A. (2011). Disordered iron homeostasis in chronic heart failure: prevalence, predictors, and relation to anemia, exercise capacity, and survival. J. Am. Coll. Cardiol..

[7] Zhou X., Xu W., Xu Y., Qian Z. (2019). Iron supplementation improves cardiovascular outcomes in patients with heart failure. Am. J. Med..

[8] von Haehling S., Ebner N., Evertz R., Ponikowski P., Anker S.D. (2019). Iron deficiency in heart failure: an overview. JACC Heart Fail..

[9] Yancy C.W., Jessup M., Bozkurt B. (2017). 2017 ACC/AHA/HFSA Focused update of the 2013 ACCF/AHA guideline for the management of heart failure: a report of the American College of Cardiology/American Heart Association Task Force on Clinical Practice Guidelines and the Heart Failure Society of America. J. Am. Coll. Cardiol..

[10] Ponikowski P., Voors A.A., Anker S.D. (2016). 2016 ESC Guidelines for the diagnosis and treatment of acute and chronic heart failure: The Task Force for the diagnosis and treatment of acute and chronic heart failure of the European Society of Cardiology (ESC)Developed with the special contribution of the Heart Failure Association (HFA) of the ESC. Eur. Heart J..

[11] Ponikowski P., van Veldhuisen D.J., Comin-Colet J. (2015). Beneficial effects of long-term intravenous iron therapy with ferric carboxymaltose in patients with symptomatic heart failure and iron deficiency†. Eur. Heart J..

[12] Anker S.D., Comin Colet J., Filippatos G., Willenheimer R., Dickstein K., Drexler H., Lüscher T.F., Bart B., Banasiak W., Niegowska J., Kirwan B.A., Mori C., von Eisenhart Rothe B, Pocock S.J., Poole-Wilson P.A., Ponikowski P., FAIR-HF Trial Investigators (2009). Ferric carboxymaltose in patients with heart failure and iron deficiency. N. Engl. J. Med..

[13] Ponikowski P., Kirwan B.A., Anker M.S. (2020). Ferric carboxymaltose for iron deficiency at discharge after acute heart failure: a multicentre randomised, controlled, double-blind trial. Lancet.

[14] Anker S.D., Comin Colet J., Filippatos G. (2009). Ferric carboxymaltose in patients with heart failure and iron deficiency. N. Engl. J. Med..

[15] Liberati A., Altman D.G., Tetzlaff J. (2009). The PRISMA statement for reporting systematic reviews and meta-analyses of studies that evaluate healthcare interventions: explanation and elaboration. Bmj.

[16] Sterne J.A.C., Savović J., Page M.J. (2019). RoB 2: a revised tool for assessing risk of bias in randomised trials. BMJ.

[17] Borenstein M., Hedges L.V., Higgins J.P.T. (2010). A basic introduction to fixed-effect and random-effects models for meta-analysis. Res. Synthesis Methods.

[18] Addressing Reporting Biases. Cochrane Handbook for Systematic Reviews of Interventions, 2008, pp. 297–333.

[19] Higgins J.P., Thompson S.G., Deeks J.J. (2003). Measuring inconsistency in meta-analyses. Bmj.

[20] Okonko D.O., Grzeslo A., Witkowski T. (2008). Effect of intravenous iron sucrose on exercise tolerance in anemic and nonanemic patients with symptomatic chronic heart failure and iron deficiency FERRIC-HF: a randomized, controlled, observer-blinded trial. J. Am. Coll. Cardiol..

[21] Toblli J.E., Lombrana A., Duarte P., Di Gennaro F. (2007). Intravenous iron reduces NT-pro-brain natriuretic peptide in anemic patients with chronic heart failure and renal insufficiency. J. Am. Coll. Cardiol..

[22] Arutyunov G., Bylova N., Ivleva A., Kobalava Z. (2009). The safety of intravenous (IV) ferric carboxymaltose versus IV iron sucrose in patients with chronic heart failure (CHF) and chronic kidney disease (CKD) with iron deficiency (ID). Eur. J. Heart Fail. Suppl..

[23] McCullough P.A., Uhlig K., Neylan J.F., Pergola P.E., Fishbane S. (2018). Usefulness of oral ferric citrate in patients with iron-deficiency anemia and chronic kidney disease with or without heart failure. Am. J. Cardiol..

[24] Beck-da-Silva L., Piardi D., Soder S., Rohde L.E., Pereira-Barretto A.C., de Albuquerque D., Bocchi E., Vilas-Boas F., Moura L.Z., Montera M.W., Rassi S., Clausell N. (2013). IRON-HF study: a randomized trial to assess the effects of iron in heart failure patients with anemia. Int J Cardiol..

[25] Savarese G., Lund L.H. (2017). Global public health burden of heart failure. Card Fail. Rev..

[26] Jackson S.L., Tong X., King R.J. (2018). National Burden of Heart Failure Events in the United States, 2006 to 2014. Circulation: Heart Fail..

[27] Cowie M.R., Anker S.D., Cleland J.G.F. (2014). Improving care for patients with acute heart failure: before, during and after hospitalization. ESC Heart Fail..

[28] Drexler H., Fuchs M. (2002). Current limitations in treatment of heart failure: New avenues and treatment options. J. Cardiovasc. Electrophysiol..

[29] Vijayakumar S., Vaduganathan M., Butler J. (2018). Glucose-lowering therapies and heart failure in type 2 diabetes mellitus: mechanistic links, clinical data, and future directions. Circulation.

[30] Greene S.J., DeVore A.D. (2020). The maximally tolerated dose: the key context for interpreting subtarget medication dosing for heart failure. JACC Heart Fail..

[31] Greene S.J., Fonarow G.C., DeVore A.D. (2019). Titration of medical therapy for heart failure with reduced ejection fraction. J. Am. Coll. Cardiol..

[32] Eivazi M., Mutharasan R.K., Cleveland E. (2019). Outcome of titrating guideline directed medical therapy in heart failure patients at 90-day post-hospital discharge. J. Cardiac Fail..

[33] Heidenreich P.A., Hernandez A.F., Yancy C.W. (2012). Get with the Guidelines program participation, process of care, and outcome for Medicare patients hospitalized with heart failure. Circ. Cardiovasc. Qual. Outcomes.

[34] Anker S.D., Kirwan B.A., van Veldhuisen D.J. (2018). Effects of ferric carboxymaltose on hospitalisations and mortality rates in iron-deficient heart failure patients: an individual patient data meta-analysis. Eur. J. Heart Fail..

[35] Luthi J.C., Flanders W.D., Burnier M., Burnand B., McClellan W.M. (2006). Anemia and chronic kidney disease are associated with poor outcomes in heart failure patients. BMC Nephrol..

[36] Virani S.A., Khosla A., Levin A. (2008). Chronic kidney disease, heart failure and anemia. Can. J. Cardiol..

[37] Hörl W.H. (2007). Iron therapy for renal anemia: how much needed, how much harmful?. Pediatr. Nephrol..

[38] Lentini S., Kaiser A., Kapsa S., Matsuno K., van der Mey D. (2020). Effects of oral iron and calcium supplement on the pharmacokinetics and pharmacodynamics of molidustat: an oral HIF-PH inhibitor for the treatment of renal anaemia. Eur. J. Clin. Pharmacol..

[39] Charles-Edwards G., Amaral N., Sleigh A., Ayis S., Catibog N., McDonagh T., Monaghan M., Amin-Youssef G., Kemp G.J., Shah A.M., Okonko D.O. (2019). Effect of iron isomaltoside on skeletal muscle energetics in patients with chronic heart failure and iron deficiency. Circulation.

[40] Comin-Colet J., Lainscak M., Dickstein K., Filippatos G.S., Johnson P., Lüscher T.F., Mori C., Willenheimer R., Ponikowski P., Anker S.D., The effect of intravenous ferric carboxymaltose on health-related quality of life in patients with chronic heart failure and iron deficiency: A subanalysis of the FAIR-HF study. (2013). 10.1093/eurheartj/ehr504 PMC3533918 22297124

[41] van Veldhuisen D.J., Ponikowski P., van der Meer P., Metra M., Böhm M., Doletsky A., Voors A.A., Macdougall I.C., Anker S.D., Roubert B., Zakin L., Cohen-Solal A., Investigators E.F.F.E.C.T.-H.F. (2017). Effect of ferric carboxymaltose on exercise capacity in patients with chronic heart failure and iron deficiency. Circulation.

[42] Lewis G.D., Malhotra R., Hernandez A.F., McNulty S.E., Smith A., Felker G.M., Tang W.H.W., LaRue S.J., Redfield M.M., Semigran M.J., Givertz M.M., Van Buren P., Whellan D., Anstrom K.J., Shah M.R., Desvigne-Nickens P., Butler J., Braunwald E. (2017). NHLBI heart failure clinical research network. effect of oral iron repletion on exercise capacity in patients with heart failure with reduced ejection fraction and iron deficiency: the IRONOUT HF randomized clinical trial. JAMA.

