# Supplementary material for: Effect of iron supplementation in patients with heart failure and iron deficiency: A systematic review and meta-analysis
Source: Int J Cardiol Heart Vasc. 2021 Sep 14;36:100871. doi: 10.1016/j.ijcha.2021.100871 (PMC8450242; doi:10.1016/j.ijcha.2021.100871)

**Supplementary Appendix**

**Table S1:** Search strategy used in each database

**Figure S1:** Risk of bias summary for included trials

**Table S1:** Search strategy used in each database

| Database | Search strategy | Articles retrieved |
| --- | --- | --- |
| Medline | ("iron"[MeSH Terms] OR "iron"[All Fields] OR ("ferric carboxymaltose"[Supplementary Concept] OR "ferric carboxymaltose"[All Fields]) OR ("ferric oxide, saccharated"[MeSH Terms] OR ("ferric"[All Fields] AND "oxide"[All Fields] AND "saccharated"[All Fields]) OR "saccharated ferric oxide"[All Fields] OR ("iron"[All Fields] AND "sucrose"[All Fields]) OR "iron sucrose"[All Fields]) OR (("mouth"[MeSH Terms] OR "mouth"[All Fields] OR "oral"[All Fields]) AND ("iron"[MeSH Terms] OR "iron"[All Fields]))) AND ("heart failure"[MeSH Terms] OR ("heart"[All Fields] AND "failure"[All Fields]) OR "heart failure"[All Fields] OR "HF"[All Fields]) | 2201 |
| Cochrane CENTRAL | (iron OR ferric carboxymaltose OR FCM OR iron sucrose) AND (heart failure OR CHF OR AHF OR HF) | 398 |

**Figure S1:** Risk of bias summary for included trials


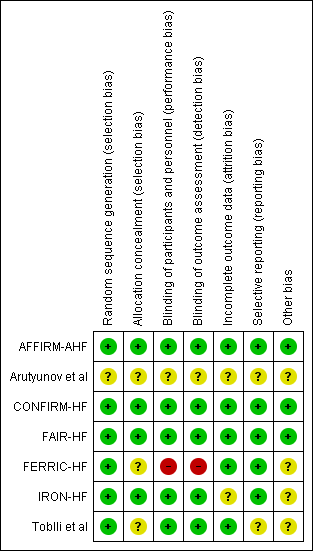

Supplement: Supplementary data 1 [file mmc1.docx]
